# Setting up a quantitative SPECT imaging network for a European multi-centre dosimetry study of radioiodine treatment for thyroid cancer as part of the MEDIRAD project

**DOI:** 10.1186/s40658-020-00332-9

**Published:** 2020-10-08

**Authors:** Jan Taprogge, Francesca Leek, Tino Schurrat, Johannes Tran-Gia, Delphine Vallot, Manuel Bardiès, Uta Eberlein, Michael Lassmann, Susanne Schlögl, Alex Vergara Gil, the MEDIRAD WP3 Investigator Team, Andreas Buck, Naomi Clayton, Frédéric Courbon, Constantin Lapa, Markus Luster, Erick Mora-Ramirez, Kate Newbold, Sarah Schumann, Frederik Verburg, Lavinia Vija, Slimane Zerdoud, Glenn D. Flux

**Affiliations:** 1 Joint Department of Physics, Royal Marsden NHSFT, Downs Road, Sutton, SM2 5PT UK; 2 The Institute of Cancer Research, 123 Old Brompton Road, London, SW7 3RP UK; 3 Department of Nuclear Medicine, Philipps-University Marburg, Baldingerstrasse, 35043 Marburg, Germany; 4 Department of Nuclear Medicine, University of Würzburg, Oberdürrbacher Str. 6, 97080 Würzburg, Germany; 5 IUCT Oncopole, Av. Irène Joliot-Curie, 31100 Toulouse, France; 6 Centre de Recherches en Cancérologie de Toulouse, UMR 1037, INSERM, Université Paul Sabatier, Toulouse, France

**Keywords:** Multi-centre trial, Dosimetry, Radioiodine, Differentiated thyroid cancer

## Abstract

**Background:**

Differentiated thyroid cancer has been treated with radioiodine for almost 80 years, although controversial questions regarding radiation-related risks and the optimisation of treatment regimens remain unresolved. Multi-centre clinical studies are required to ensure recruitment of sufficient patients to achieve the statistical significance required to address these issues. Optimisation and standardisation of data acquisition and processing are necessary to ensure quantitative imaging and patient-specific dosimetry.

**Material and methods:**

A European network of centres able to perform standardised quantitative imaging of radioiodine therapy of thyroid cancer patients was set-up within the EU consortium MEDIRAD. This network will support a concurrent series of clinical studies to determine accurately absorbed doses for thyroid cancer patients treated with radioiodine. Five SPECT(/CT) systems at four European centres were characterised with respect to their system volume sensitivity, recovery coefficients and dead time.

**Results:**

System volume sensitivities of the Siemens Intevo systems (crystal thickness 3/8″) ranged from 62.1 to 73.5 cps/MBq. For a GE Discovery 670 (crystal thickness 5/8″) a system volume sensitivity of 92.2 cps/MBq was measured. Recovery coefficients measured on three Siemens Intevo systems show good agreement. For volumes larger than 10 ml, the maximum observed difference between recovery coefficients was found to be ± 0.02. Furthermore, dead-time coefficients measured on two Siemens Intevo systems agreed well with previously published dead-time values.

**Conclusions:**

Results presented here provide additional support for the proposal to use global calibration parameters for cameras of the same make and model. This could potentially facilitate the extension of the imaging network for further dosimetry-based studies.

## Background

Radioiodine ([^131^I]NaI) has been used to treat thyroid cancer following partial or total thyroidectomy for nearly 80 years. Nevertheless, treatment regimens remain subject to controversy and administered activities can vary widely, in part due to a lack of evidence regarding potential risks from treatment. A consensus paper [1] developed by experts from the American Thyroid Association (ATA) [2], the European Association of Nuclear Medicine (EANM), the Society of Nuclear Medicine and Molecular Imaging (SNMMI) and the European Thyroid Association (ETA) has established several principles regarding treatment and has highlighted areas in need of investigation. These ‘Martinique principles’ include the need to determine the optimal prescribed activity of radioiodine for adjuvant treatment and for patients at low risk. Furthermore, they recommend that major gaps in knowledge concerning the optimal use of radioiodine should be addressed by prospective studies.

A review by Hackshaw et al. [3] concluded that it is not possible to determine from the literature whether ablation success rates are higher with higher administered activities. Several authors have hypothesised that the ablation success rate is dependent on the absorbed dose delivered to residual thyroid tissue rather than on the activity administered [4–7]. A number of studies have shown that a large range of absorbed doses is observed in patients when empirical activities are used [4–12]. Previous studies that have investigated the relationship between the absorbed dose to the thyroid remnant and the treatment success rate have not been performed in a multi-centre setting, and treatment based on a dosimetry approach has, therefore, not been widely adopted. Multi-centre prospective clinical studies are ultimately necessary to resolve the controversies raised in the consensus paper [1] by the ATA, EANM, SNMMI and ETA.

Clinical studies performed in a multi-centre setting enable a wider input into the trial design and data analysis [13]. A summary of the physics aspects of setting up a multi-centre clinical trial involving imaging-based dosimetry has been provided in [14]. Multi-centre clinical studies involving a dosimetry component must be carefully planned and a consistent approach to quality assurance should be implemented to allow for the collation of results from the individual centres. Image data acquisition and processing can only be standardised to a certain level due to local differences in logistics, available equipment and constraints in ethics approvals and regulations.

Quantitative imaging is becoming more widely adopted [15] and will in future be part of many multi-centre clinical studies in nuclear medicine. Guidelines for quantitative imaging of ^131^I have been provided by the Committee on Medical Internal Radiation Dose (MIRD) in pamphlet 24 [16]. The EANM has issued guidelines on internal dosimetry for ^131^I[mIBG] treatment of neuroendocrine tumours [17].

Multi-centre studies with a dosimetry component require the set-up of a network of cameras able to perform quantitative imaging [14]. Zimmerman et al. [18] set up a multi-national, multi-centre phantom study to evaluate accuracy and reproducibility of SPECT image quantification with ^133^Ba as a surrogate for ^131^I. Wevrett et al. [19] assessed the feasibility of using an international inter-comparison exercise for ^177^Lu as a means to ensure consistency between clinical sites. A study in the Netherlands [20] reported on the variability in ^177^Lu SPECT quantification between different state-of-the-art SPECT/CT systems. Peters et al. [21] investigated the quantitative accuracy and inter-system variability of various SPECT/CT systems with phantom measurements using ^99m^Tc in a multi-vendor and multi-centre setting. Dickson et al. [22] proposed a framework for DaTSCAN ([^123^I]FP-CIT) imaging standardisation. An example of a multi-centre clinical trial involving dosimetry for [^123^I]NaI and [^131^I]NaI is SEL-I-METRY [23], a phase II clinical trial using quantitative SPECT imaging to investigate the potential of selumetinib to resensitise advanced iodine refractory differentiated thyroid cancer (DTC) to radioiodine. As part of SELIMETRY, a quantitative SPECT imaging network was set up in the UK [24].

MEDIRAD is a European Horizon 2020 funded project investigating the implications of medical low dose radiation exposure [25]. The overall objectives of MEDIRAD Work Package 3 (WP3) are to develop and implement the tools necessary to, for the first time in a multi-centre setting, investigate the range of absorbed doses delivered to healthy organs in patients undergoing thyroid ablation and to establish a threshold absorbed dose required for a successful ablation. Absorbed dose estimates to the thyroid remnant will be used to investigate the relationship between the radiation dose to the remnant tissue and treatment success. A sub-task of WP3 is to assess the variation between patient biokinetics, the success of ablation and the occurrence of short to mid-term toxicities. This is a concurrent series of non-randomised, non-blinded, prospective observational studies aiming to recruit 100 patients across four centres (Royal Marsden Hospital, Universitätsklinikum Marburg, Universitätsklinikum Würzburg and Institute Universitaire du Cancer de Toulouse Oncopole). A series of SPECT/(CT) (hereafter referred to as SPECT(/CT)) and whole-body scans is performed following radioiodine therapy from 6 to 168 h post-administration to perform centralised dosimetry calculations for thyroid remnants, healthy organs and metastases. Whole-body (WB) retention measurements and, for a sub-group of patients, blood samples are collected to enable the calculation of WB and blood absorbed doses. Patients are followed up at regular clinical visits to assess the success of ablation and discover short to mid-term toxicity.

The aim of this work was to develop the required methodologies and perform the gamma camera performance assessments necessary for the set-up of a European imaging network for quantitative [^131^I]NaI imaging to support a concurrent series of dosimetry-based clinical studies for radioiodine therapy of thyroid cancer patients. A degree of flexibility was required to enable the set-up of these centres due to differences in local logistics and in the interpretation of radiation protection legislation in the four participating centres located in the three countries. Local radiation protection restrictions prevented the use of large amounts of liquid [^131^I]NaI to determine dead-time of the systems.

## Methods

### Setting up a network of gamma cameras for quantitative SPECT imaging

The four centres involved in the MEDIRAD study were equipped with a total of 5 SPECT(/CT) systems which are summarised in Table 1. All SPECT(/CT) systems were calibrated for quantitative high activity radioiodine imaging by performing pre-study site visits involving measurements to determine system volume sensitivity, recovery coefficients and dead-time characteristics for each SPECT(/CT) system used for the study.

**Table 1:** Summary of the imaging systems used for the MEDIRAD clinical study

System	Centre	Crystal thickness	Reconstruction software*	Attenuation correction**
Siemens Symbia S	Centre A	3/8″	Flash 3D	Chang
Siemens Intevo (1)	Centre B	3/8″	Flash 3D	CT
Siemens Intevo (2)	Centre B	3/8″	Flash 3D	CT
Siemens Intevo Bold	Centre C	3/8″	Flash 3D	CT
GE Discovery 670	Centre D	5/8″	Volumetrix MI	CT

*Vendor-specific reconstruction software/algorithm

**Attenuation correction method used for present study

The system volume sensitivity characterises the system’s response to a uniform concentration of activity. SPECT recovery coefficients, defined as the ratio between the observed activity concentration in tomographic imaging and the true activity concentration [26], were determined to correct for partial volume and resolution effects on the activity concentration measured in the reconstructed SPECT images. Dead-time factors, defined as the ratio between the true count-rate and the observed count-rate of a detector, are used to correct the acquired image counts for counts lost due to detector paralysis when imaging high activities of ^131^I.

Prior to the site set-up measurements, it was ensured that each centre had performed the following routine quality control tests [27] according to local limits; photopeak position, ^131^I and/or ^99m^Tc intrinsic uniformity, centre of rotation for high-energy collimators used in the study, SPECT/CT system alignment, extrinsic high-energy collimator flood, QC of weighing scales used in these measurements and QC of dose calibrators used in these measurements.

Activities used for the phantom measurements were measured with dose calibrators that were traceable to a national standard, had been calibrated using an accredited laboratory for calibration in the respective countries or was calibrated to a local standard (e.g. a calibrated high purity germanium detector).

### Whole-body and SPECT acquisition and reconstruction protocols

Standardised SPECT acquisition and reconstruction protocols were used on all systems involved in the study for the site set-up measurements and all patient measurements. Triple-energy scatter correction was used on all systems. CT attenuation correction was performed using the local standard low-dose CT protocol. As no hybrid SPECT/CT system was available for one centre, the Chang attenuation correction [28] was applied for this centre. ^131^I acquisition and reconstruction parameters are summarised in Tables 2 and 3. All SPECT/CT reconstructions included resolution recovery.

**Table 2:** Acquisition parameters used for ^131^I imaging as part of the MEDIRAD WP3 study

Parameter	^131^I acquisition protocol
Collimator	High Energy (HE)
Photopeak-energy window	364 keV ± 10%
Lower scatter-energy window	318 keV ± 3%
Higher scatter-energy window	413 keV ± 3%
WB planar-acquisition mode	Continuous movement at 20 cm/min
SPECT(/CT) matrix	128 × 128
SPECT movement	Body contour
Projections	2 × 30 (6° projection)
Time per projection	Adjusted based on measured count-rate for patient acquisition
CT	Standard low-dose protocol

**Table 3:** SPECT(/CT) reconstruction parameters used for ^131^I imaging as part of the MEDIRAD WP3 study

Parameter	^131^I reconstruction protocol
Reconstruction	OSEM (4 iterations, 10 subsets)
Attenuation correction (AC)	CTAC (one site: Chang with 0.11 cm^−1^ @ 364 keV)
Scatter correction	Triple-energy window (TEW)
Post-reconstruction filtering	None

### System volume sensitivity measurement

A cylindrical or body-shaped phantom with a volume greater than 6 l was used for all system volume sensitivity measurements based on local availability of phantoms. The volume of each phantom was accurately determined by measuring the weight of water needed to completely fill the phantom. In total, 40 ± 2 MBq of liquid [^131^I]NaI, 1 g of potassium iodine and 1 g of sodium thiosulphate were added to the phantom. The activity of 40 MBq was chosen to minimise the influence of dead-time of the SPECT systems on the measurements. The activity was measured accurately using a radionuclide calibrator. An acquisition of 100 kilo counts (kcounts) per SPECT projection was performed using the parameters in Table 2 and the image was reconstructed locally at each centre using the SPECT reconstruction parameters listed in Table 3.

The system volume sensitivity *Q*_*vol*_ in counts-per-second per MBq (cps/MBq) was obtained from placing a 15 cm diameter volume-of-interest (VOI) in the centre of the reconstructed SPECT image of the phantom and was defined as:

1 $$ {Q}_{vol}=\frac{C_{VOI}}{a_{\mathrm{conc}}^{\mathrm{mid}}\bullet {V}_{VOI}\bullet \frac{\#P}{2}\bullet {P}_{\mathrm{time}}} $$

where *C*_*VOI*_ is the number of counts in the 15 cm VOI, ${a}_{\mathrm{conc}}^{\mathrm{mid}}$ is the activity concentration in the phantom decay corrected to the mid-point of the scan in MBq/ml, *V*_*VOI*_ is the volume of the 15 cm VOI in ml, #*P* is the number of projections, and *P*_*time*_ is the time per projection in seconds. The division of #*P* by a factor of 2 originates from the 2 detectors that were available for all SPECT(/CT) systems.

Uncertainty analysis was performed following recent EANM guidance [29]. Uncertainty in *Q*_*vol*_, *u*(*Q*_*vol*_), was estimated as:

2 $$ \frac{u\left({Q}_{vol}\right)}{Q_{vol}}=\frac{u\left({a}_{\mathrm{conc}}^{\mathrm{mid}}\right)}{a_{\mathrm{conc}}^{\mathrm{mid}}} $$

*C*_*VOI*_, *V*_*VOI*_, #P, *P*_*time*_ were assumed to have no associated measurement uncertainty. Uncertainty of ${a}_{\mathrm{conc}}^{\mathrm{mid}}$, $u\left({a}_{\mathrm{conc}}^{\mathrm{mid}}\right)$, was calculated as:

3 $$ {\left(\frac{u\left({a}_{\mathrm{conc}}^{\mathrm{mid}}\right)}{a_{\mathrm{conc}}^{\mathrm{mid}}}\right)}^2={\left(\frac{u\left({A}_{mid}\right)}{A_{mid}}\right)}^2+{\left(\frac{u\left({V}_{phantom}\right)}{V_{phantom}}\right)}^2 $$

where *A*_*mid*_ is the activity in MBq measured on the dose calibrator decay corrected to the mid-point of the scan and *u*(*A*_*mid*_) is the uncertainty in the dose calibrator measurement. *u*(*A*_*mid*_) was taken to be the measurement uncertainty provided on the calibration certificates for the respective calibrators or was assumed to be ± 5% where the measurement uncertainty was unknown. This is the acceptable calibration tolerance for field instruments in the UK [30]. *V*_*phantom*_ is the phantom volume in millilitre. The uncertainty in the phantom volume *u*(*V*_*phantom*_) was estimated to be ± 5 ml due to the potential for small air bubbles in the filled phantom.

### SPECT recovery coefficient determination

**Fig. 1:**
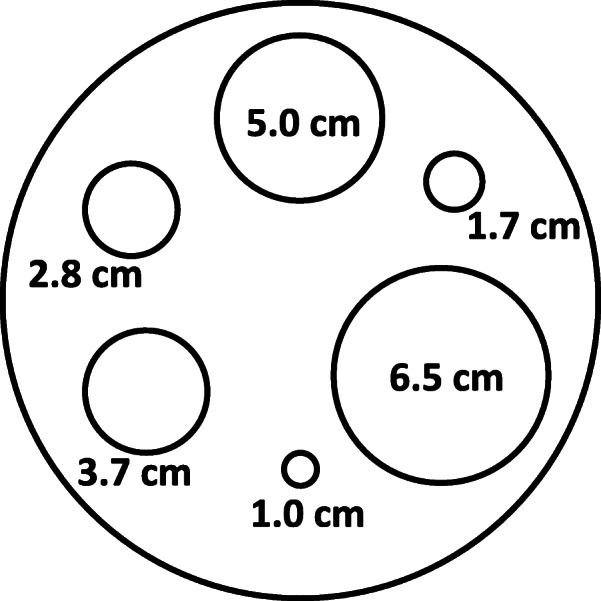
Schematic representation of the placement of the six spheres used for the recovery coefficient determination in a cylindrical IEC head phantom

A cylindrical IEC head phantom (inner diameter 19.7 cm, inner height 18.3 cm) was used for the recovery coefficient measurements. A custom-designed lid with six 3D-printed sphere inserts was used with internal diameters of 1.0, 1.7, 2.8, 3.7, 5.0 and 6.5 cm (Fig. 1). Internal volumes of all spheres were obtained by measuring the weight difference of empty and filled spheres. The spheres were filled with a solution of water, [^131^I]NaI, potassium iodide and sodium thiosulphate. The ^131^I activity concentration in the spheres was specified to lie in the range of 0.5-0.6 MBq/ml at the time of acquisition. This is the expected maximum activity concentration in salivary glands estimated from published maximum uptake values by Liu et al. [31]. MEDIRAD is investigating the implications of medical low dose radiation exposure and the salivary glands are one of the organs-at-risk of particular interest in radioiodine therapy. The background compartment of the phantom was filled with water only. SPECT acquisitions of the phantom with 60 s per view and the parameters detailed in Table 2 were performed and reconstructed locally using the reconstruction parameters in Table 3.

Spherical VOIs matching the nominal dimensions of the spheres were drawn on the CT. After interpolation of VOIs from CT to SPECT matrix size, the VOIs were copied to the reconstructed SPECT image. For the centre with no access to a hybrid SPECT/CT system, those VOIs were drawn on the reconstructed SPECT image. Recovery coefficients ${R}_c^{sphere}$ for each sphere were calculated as:

4 $$ {R}_c^{\mathrm{sphere}}=\frac{C_{\mathrm{sphere}}}{a_{\mathrm{conc}}^{\mathrm{mid}}\bullet {V}_{\mathrm{sphere}}\bullet \frac{\#P}{2}\bullet {P}_{\mathrm{time}}}\bullet \frac{1}{Q_{\mathrm{vol}}} $$

Here, *C*_sphere_ is the number of counts in a sphere, ${a}_{\mathrm{conc}}^{\mathrm{mid}}$ is the activity concentration in the same sphere decay-corrected to the mid-point of the scan in MBq/ml, *V*_sphere_ is the volume of the sphere in millilitre, #*P* is the number of projections and *P*_time_ is the time per projection in seconds. *Q*_vol_ is the system volume sensitivity of the respective system. The division of #*P* by a factor of 2 originates from the 2 detectors that were available for all SPECT(/CT) systems.

Recovery coefficients for each sphere ${R}_c^{\mathrm{sphere}}$ were plotted against sphere volume *V*_sphere_ and a recovery curve fitted using gnuplot version 5.2.7. The fitted recovery curve was defined as:

5 $$ {R}_c(V)={R}_{\mathrm{plateau}}-\frac{R_{\mathrm{plateau}}}{1+{\left(\frac{V}{\beta}\right)}^{\gamma }} $$

With *V* as the volume in millilitre, and *β*, *γ* and *R*_plateau_ are fit parameters. Parameter error estimates were obtained from gnuplot version 5.2.7 as the asymptotic standard errors.

To compare the recovery curves of different systems, the maximum observed absolute difference in the fitted recovery factor $\Delta  {R}_c^{\mathrm{max}}$ for a given volume was calculated as:

6 $$ \Delta  {R}_c^{\mathrm{max}}=\operatorname{Max}\left(\left|{R}_c\left(V,\mathrm{System}\ 1\right)-{R}_c\left(V,\mathrm{System}\ 2\right)\right|\right) $$

With *R*_*c*_(*V*, System 1) and *R*_*c*_(*V*, System 2) as the recovery factors of the two systems to be compared for a given volume *V*.

### Dead-time characterisation

Dead-time measurements were performed using a 3700 MBq ^131^I capsule placed in a cylindrical scatter phantom made from polymethyl methacrylate (PMMA) developed at the Royal Marsden Hospital (Sutton, UK). The phantom had a diameter of 13 cm and a height of 13 cm to represent a typical neck size, with a 2.5-cm-diameter hole in the middle extending from the top of the phantom to the centre for inserting the ^131^I capsule.

A series of static planar scans was acquired whilst the capsule was decaying. Measurements were performed approximately every second day until the capsule had decayed to 1 GBq and thereafter measurements were performed every 3-4 days. Each acquisition encompassed a static planar scan of 100 kcounts for each detector head with the capsule in the centre of the scatter phantom. Acquisition times per detector head were extracted from the DICOM headers to calculate the observed count rate. The phantom was placed on the patient bed at approximately 10 cm from the detector surface. Additionally, 10-min background acquisitions were performed with no source in place to correct for background activity.

Measurements with capsule activities below 100 MBq were assumed to be unaffected by dead-time and were used to determine the relationship between true count-rate ${\dot{C}}_{\mathrm{true}}$ and source activity level from a linear fit of background corrected count rates versus source activities.

Dead-time correction factors for each measurement were determined as:

7 $$ DF\left({\dot{C}}_{\mathrm{true}}\right)=\frac{{\dot{C}}_{\mathrm{true}}}{{\dot{C}}_{\mathrm{observed}}} $$

Where ${\dot{C}}_{\mathrm{observed}}$ is the background-corrected measured count-rate of the detector head at each source activity level.

Dead-time *τ* was obtained from a fit using gnuplot version 5.2.7 of a non-paralysable detector model:

8 $$ DF\left({\dot{C}}_{\mathrm{true}}\right)=\frac{1}{\left(1-\uptau \bullet {\dot{C}}_{\mathrm{observed}}\right)} $$

Parameter error estimates were obtained from gnuplot version 5.2.7 as the asymptotic standard errors.

On two systems, Intevo 1 and Intevo 2, the methodology used here was validated against the dead-time measurement methodology presented by Gregory et al. [24]. The authors determined dead time by incrementally adding ^131^I to a Jaszczak phantom and performing 100 kcounts static images for each activity level.

No dead-time measurements were performed on the GE Discovery 670 as at the respective centre imaging will only be performed at imaging time points later than 48 h after the radioiodine administration. It is assumed that the activity level at such late imaging time points is low enough to ignore dead-time effects.

## Results

### System volume sensitivity

The system volume sensitivity values for the five systems are presented in Table 4. The system volume sensitivity value of the SPECT-only Siemens Symbia S was obtained using the Chang attenuation correction. System volume sensitivity of the Intevo Bold system was found to be approximately 18% higher compared to the two Intevo systems.

**Table 4:** System volume sensitivity values for the five systems used in the MEDIRAD WP3 study

System	System volume sensitivity [cps/MBq]
Siemens Intevo (1)	63.0 ± 1.9
Siemens Intevo (2)	62.1 ± 1.9
Siemens Intevo Bold	73.5 ± 3.7
Siemens Symbia S*	55.6 ± 3.0
GE Discovery 670	92.2 ± 2.8

*Chang’s attenuation correction

### SPECT recovery coefficient

**Fig. 2:**
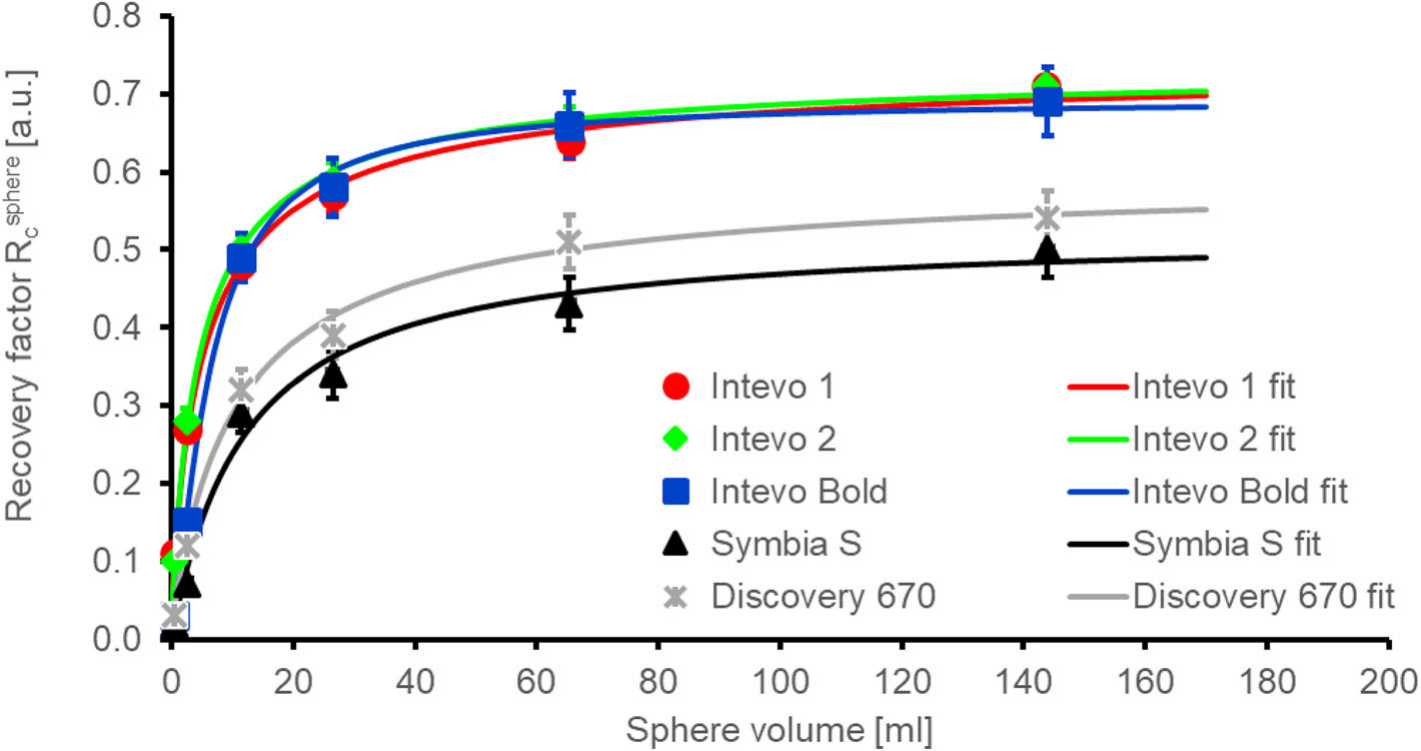
Measured recovery coefficients (in arbitrary units—a.u.) for the five systems assessed here and the respective fits using Eq. (5)

The recovery coefficients of the five systems are shown in Fig. 2 together with the respective fits using Eq. (5) in gnuplot version 5.2.7. A good agreement is observed between measured recovery coefficients and fits. The obtained fit parameters are summarised in Table 5.

**Table 5:** Fit parameters for the recovery curve fitted using Eq. (5)

System	Recovery curve fit parameters		
	*R*_plateau_	*β*	*γ*
Siemens Intevo (1)	0.74 ± 0.03	5.42 ± 0.77	0.82 ± 0.07
Siemens Intevo (2)	0.73 ± 0.02	4.75 ± 0.37	0.88 ± 0.04
Siemens Intevo Bold	0.72 ± 0.02	6.34 ± 0.48	1.15 ± 0.10
Siemens Symbia S	0.52 ± 0.05	11.95 ± 3.43	1.02 ± 0.23
GE Discovery 670	0.60 ± 0.03	10.75 ± 1.45	0.91 ± 0.07

The recovery curves for the Intevo Bold, Intevo 1 and Intevo 2 are similar whilst the recovery curve of the Symbia S is lower. The maximum observed absolute differences in the recovery curves, $\Delta  {R}_c^{\mathrm{max}}$, for Intevo 1 and Intevo 2 were found to be 0.022 for a volume of 12 ml. For volumes larger than 10 ml, the two systems Intevo 1 and Intevo Bold have a similar $\Delta  {R}_c^{\mathrm{max}}$ of 0.018, indicating a good agreement between the curves. Nevertheless, for volumes smaller than 10 ml, the recovery curves of Intevo 1 and Intevo Bold vary by up to an $\Delta  {R}_c^{\mathrm{max}}$ of 0.103. Results for the Discovery 670 show that the recovery curve is lower than that of Intevo Bold and Intevo 1/2.

### Dead-time factor

**Fig. 3:**
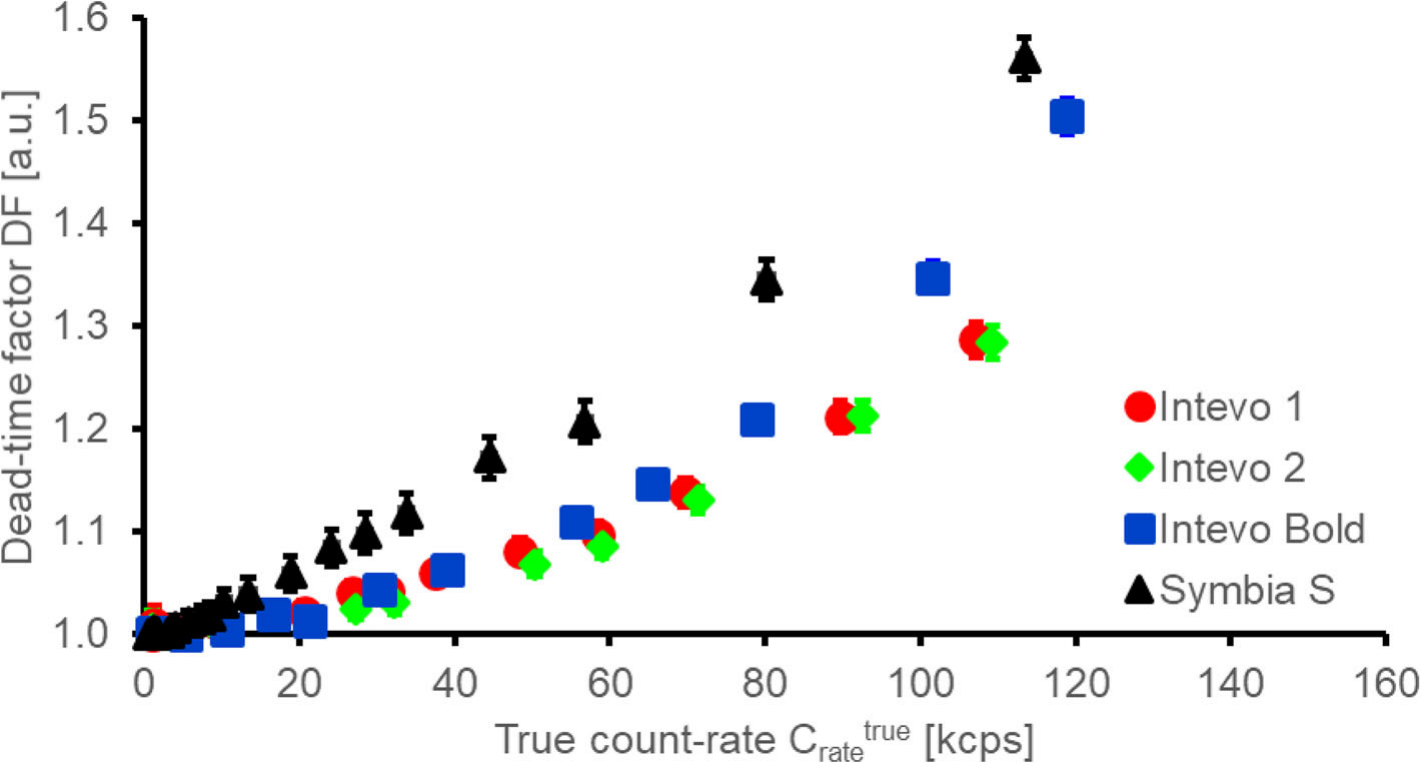
Dead-time factor (in arbitrary units—a.u.) of the four Siemens systems used for the clinical part of the MEDIRAD study shown as a function of true count-rate of the system

In Fig. 3, dead-time factors for the four systems that were used for high-activity ^131^I imaging in MEDIRAD are plotted against the true count-rate of the system. The maximum activity imaged of approximately 3700 MBq resulted in true count-rates of 115 ± 10 kilo counts-per-second (kcps) on the four systems. An activity of 1000 MBq in the field-of-view (FOV) was associated with true count rates of 30 ± 3 kcps.

**Fig. 4:**
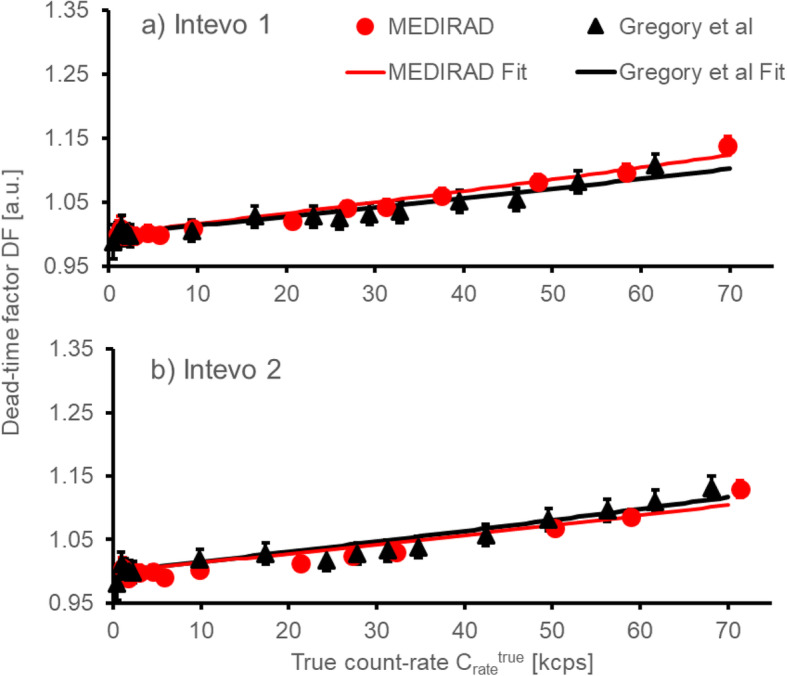
Comparison of the dead-time factors (in arbitrary units—a.u.) measured using the methodology by Gregory et al. [24] and the MEDIRAD methodology. The corresponding fits using a non-paralysable detector model (Eq. (8)) are shown as red and black lines, respectively

Figure 4 shows the comparison between dead-time factors obtained using the method proposed by Gregory et al. [24], which involves a series of acquisitions with increasing activity in a large volume uniform phantom, and the method used in the present study. An overall good agreement is found between the two methodologies on both systems assessed. The maximum absolute difference in dead-time factors at true count rates of up to approximately 65 kcps, corresponding to an activity of approximately 2700 MBq, was found to be ± 0.02. A fit of Eq. (8) to the dead time data of Intevo 1 measured using the methodology proposed here and the methodology used by Gregory et al. [24] resulted in dead times of 1.3 ± 0.1 and 1.5 ± 0.1 μs, respectively. For Intevo 2, dead times of 1.5 ± 0.1 and 1.4 ± 0.1 μs were obtained using the two methodologies, respectively.

## Discussion

The set-up of a quantitative imaging network, particularly involving centres in several countries, requires a certain degree of flexibility. The results presented here are for the set-up of the first European quantitative imaging network for radioiodine. Methodologies to set up a multi-national quantitative imaging network for radioiodine, which includes the assessment of system volume sensitivity, dead time and recovery coefficients were in part defined by restrictions based on the local interpretation of radiation protection laws in different countries, which for example prevented the use of large quantities of liquid radioiodine.

Due to the relatively low patient numbers in each centre, large imaging networks are required for any multi-centre clinical study aiming to recruit large numbers of patients in molecular radiotherapy. Results obtained here and by Gregory et al. [24] have provided evidence that dead-time factors are similar on gamma cameras from the same make and model. Similarly, if reconstruction protocols are standardised across the centres involved in a multi-centre study, recovery curves appear to be similar enough for matched makes and models to warrant the use of global, model-specific calibration factors as proposed by Gregory et al. [24].

In the present study, recovery curves were found to be similar for all three included Siemens SPECT/CT systems. The recovery curve of the SPECT only system is lower, potentially due to the use of Chang’s attenuation correction instead of CT attenuation correction. Differences between recovery curves of Siemens and GE systems assessed here are likely due to differences in the used high energy collimators by the two manufacturers (for comparison of septal thickness and hole length see ref. [24]), the crystal thickness of systems (3/8″ vs 5/8″) and the method of resolution recovery used in the manufacturer’s reconstruction software. The thicker crystal of the GE system is expected to result in a worse intrinsic resolution [32].

The observed difference in system volume sensitivity between the Intevo Bold and Intevo systems is a surprising result. Calculations, acquisition and reconstruction parameters were validated independently by two medical physicists. One possible explanation would be a difference in the software versions on the cameras and discussions with the manufacturer are ongoing. Further measurements on additional systems will be required to investigate this difference.

Using global calibration factors when using standardised acquisition and reconstruction parameters could potentially allow for a reduction in the site set-up measurements required before a centre can participate in a multi-centre clinical study. Acquisition and reconstruction methods are currently not standardised across centres and other studies involving molecular radiotherapy. Global calibration factors for system volume sensitivity and recovery coefficients could be determined if acquisition and reconstruction protocols would be standardised for new studies.

When using global calibration factors, validation measurements or dosimetry audits as required for external beam radiotherapy trials [33] will become more important. Those measurements range from simple sensitivity measurements to semi- or full-anthropomorphic phantoms [34–36] or testing of the full dosimetry chain [24]. In the present study, reconstructions are performed locally at the participating centres, which reduces the impact on the central dosimetry hub, but might increase site-dependent biases.

Dead-time factors measured using the methodology presented here and the one employed by Gregory et al. [24] showed good agreement within the associated uncertainties, which allows for further flexibility in future clinical studies. The methodology of Gregory et al. [24] allows for all dead-time measurements to be performed on a single day whilst the decaying source technique with a capsule of radioiodine requires measurements to be performed over several months. Nevertheless, the methodology proposed by Gregory et al. potentially leads to higher staff doses due to increased handling times of the phantom in the process of adding activity to the phantom in a step-by-step process. As multi-centre studies become more prevalent and involve centres in more countries, flexible approaches to these measurements might be required.

A limitation of the study is the small number of SPECT(/CT) systems included in the performance assessment. Two Siemens Intevo and one Siemens Intevo Bold SPECT/CT could be directly compared.

## Conclusions

The first European quantitative imaging network for high-activity radioiodine has been set-up. The imaging network will determine, for the first time in a multi-centre setting, the range of absorbed doses delivered to healthy organs in patients undergoing thyroid ablation and aims to determine the threshold absorbed dose required for a successful ablation. Results presented here for two Siemens Intevo and one Siemens Intevo Bold provide additional support for the proposal to use global calibration parameters for cameras of the same make and model. In time, we hope to find that this could simplify the extension of the imaging network to other centres. There is an urgent need to standardise the acquisition and reconstruction parameters for studies involving dosimetry in molecular radiotherapy.

## Data Availability

Data can be provided upon a reasonable request to the corresponding author.

## Abbreviations

ATA: American Thyroid Association
EANM: European Association of Nuclear Medicine
SNMMI: Society of Nuclear Medicine and Molecular Imaging
ETA: European Thyroid Association
DTC: Differentiated thyroid cancer
WP3: MEDIRAD Work Package 3
WB: Whole-body
AC: Attenuation correction
TEW: Triple-energy window
kcounts: Kilo counts
VOI: Volume-of-interest
cps/MBq: Counts-per-second per MBq
PMMA: Polymethyl methacrylate
kcps: Kilo count per second
FOV: Field-of-view
HE: High energy
